# Real-world evidence research based on big data: Motivation—challenges—success factors

**DOI:** 10.1007/s00761-018-0358-3

**Published:** 2018-06-07

**Authors:** Benedikt E. Maissenhaelter, Ashley L. Woolmore, Peter M. Schlag

**Affiliations:** 1 IQVIA (formerly Quintiles & IMS Health), Landshuter Allee 10, 80637 Munich, Germany; 2 IQVIA (formerly Quintiles & IMS Health), Paris, France; 3 c/o Charité Comprehensive Cancer Center, Berlin, Germany

**Keywords:** Real-world data, Evidence network, Network of cancer centers, Outcome research, Quality of care, Real-World-Data, Evidenznetzwerk, Netzwerk von Krebszentren, Outcome-Forschung, Versorgungsqualität

## Abstract

**Background:**

In recent years there has been an increasing, partially also critical interest in understanding the potential benefits of generating real-world evidence (RWE) in medicine.

**Objectives:**

The benefits and limitations of RWE in the context of randomized controlled trials (RCTs) are described along with a view on how they may complement each other as partners in the generation of evidence for clinical oncology. Moreover, challenges and success factors in building an effective RWE network of cooperating cancer centers are analyzed and discussed.

**Material and methods:**

This article is based on a selective literature search (predominantly 2015–2017) combined with our practical experience to date in establishing European oncology RWE networks.

**Results:**

RWE studies can be highly valuable and complementary to RCTs due to their high external validity. If cancer centers successfully address the various challenges in the establishment of an effective RWE study network and in the consequent execution of studies, they may efficiently generate high-quality research findings on treatment effectiveness and safety. Concerns pertaining to data privacy are of utmost importance and discussed accordingly. Securing data completeness, accuracy, and a common data structure on routinely collected disease and treatment-related data of patients with cancer is a challenging task that requires high engagement of all participants in the process.

**Conclusion:**

Based on the discussed prerequisites, the analysis of comprehensive and complex real-world data in the context of a RWE study network represents an important and promising complementary partner to RCTs. This enables research into the general quality of cancer care and can permit comparative effectiveness studies across partner centers. Moreover, it will provide insights into a broader optimization of cancer care, refined therapeutic strategies for patient subgroups as well as avenues for further research in oncology.

## Zusammenfassung

**Hintergrund:**

In den letzten Jahren ist ein zunehmendes, teilweise auch durchaus kritisches, Interesse an Real-World-Evidence(RWE) in der Medizin festzustellen.

**Fragestellung:**

Vor- und Nachteile von RWE sollen insbesondere im Kontext mit randomisierten klinischen Studien (RCTs) analysiert werden und dabei diskutiert werden, wie RWE und RCTs sich als komplementäre Partner zur Evidenzgenerierung in der klinischen Onkologie ergänzen können. Ferner sollen die Herausforderungen und Erfolgsfaktoren für den Aufbau eines leistungsstarken RWE-Netzwerks kooperierender Krebszentren aufgezeigt werden.

**Material und Methoden:**

Thematisch wurde die aktuelle Literatur (schwerpunktmäßig 2015–2017) selektiv recherchiert und kombiniert mit der eigenen praktischen Erfahrung aus dem bisherigen Aufbau von Europäischen Onkologischen Real-World-Evidence-Netzwerken.

**Ergebnisse:**

RWE-Studien können durch ihre hohe externe Validität eine äußerst wertvolle Ergänzung zu RCTs sein. Wenn Krebszentren die zahlreichen Herausforderungen sowohl im Aufbau eines leistungsstarken RWE-Netzwerks als auch in der späteren Studiendurchführung sorgfältig adressieren, können hochwertige Forschungsergebnisse zur Therapieeffektivität und Therapiesicherheit erzielt werden. Die mit der Nutzung vernetzter, umfangreicher und vielschichtiger Datensätze (Big Data) sich ergebenden datenschutzrechtlichen Anforderungen sind dabei a priori zu beachten. Die Sicherung qualitätsgeprüfter, angemessen vollständiger und einheitlich strukturierter onkologischer Behandlungs- sowie Verlaufsdaten ist eine anspruchsvolle Aufgabe, welche die aktive und verantwortungsvolle Mitwirkung aller hieran Beteiligten benötigt.

**Schlussfolgerungen:**

Unter den aufgezeigten Voraussetzungen stellt die Analyse umfangreicher und komplexer Real-World-Daten im Rahmen eines RWE-Netzwerks eine wichtige und vielversprechende Ergänzung zu RCTs dar. Hierdurch kann die allgemeine onkologische Versorgungsqualität analysiert und ein (Qualitäts‑)Vergleich verschiedener Einrichtungen ermöglicht werden. Zudem können auch Hinweise zu einer flächendeckenden Optimierung onkologischer Behandlung, zu verbesserten Therapiestrategien für Patientensubgruppen und Anregungen für neue onkologische Forschungsfelder gewonnen werden.

“We have entered the era of big data in healthcare” [12] and this era will transform medicine and especially oncology [13, 24]. In this article, we focus on a specific aspect: how and under which conditions can real-world evidence (RWE) enrich and improve outcome research in oncology?

## Background

The U.S. Food and Drug Administration (FDA) defines RWE as “the clinical evidence regarding the usage, and potential benefits or risks, of a medical product derived from analysis of real-world data” [34]. The British Academy of Medical Sciences employs a similar definition: “the evidence generated from clinically relevant data collected outside of the context of conventional randomised controlled trials” [33]. Common to these definitions, and others, is the focus on evidence that is clinically relevant and that stems from routine clinical practice [32]. Our understanding of *RWE is the technology-facilitated collation of all routinely collected information on patients from clinical systems to a comprehensive, homogeneously analysable dataset (big data) that reflects the treatment reality in the best possible and comparable manner.*

In recent years there has been a growing interest in the potential benefits and the relevance of RWE studies [29, 30]. For example, Tannock et al. recently pointed out in *The Lancet Oncology* that RWE studies enable “crucial insights into quality of care and effectiveness” [32]. In particular, key healthcare institutions have joined the scientific debate about when and how RWE studies can enrich our understanding of medical evidence [33, 34]. At the same time, the high value of traditional randomized controlled trials (RCTs) should not be challenged and the necessity to conduct RWE studies at a high level of methodological and scientific rigor needs to be emphasized [18].

In oncology, the American Society of Clinical Oncology (ASCO) has recently published a research statement that discusses the potential of RWE and provides recommendations on how RWE may be utilized in conjunction with RCTs [35]. We will follow this line of reasoning and assume that RWE and RCTs are principally complementary approaches in clinical research. Consequently, it follows that RWE studies can also be a valuable tool for clinical research in oncology. In the following, we first discuss the benefits of RWE studies. Subsequently, we analyze a series of specific challenges and success factors in the establishment of a RWE study network that enables relevant research studies.

## Strengths and weaknesses of RWE studies versus RCTs

Only a small proportion of cancer patients are recruited into RCTs and those that participate are typically younger and have fewer comorbidities than those that are not included into RCTs. The inclusion and exclusion criteria of RCTs usually create idealized conditions whereas per definition RWE studies provide insights into the routine clinical setting [3]. As a result, RWE studies may benefit from greater generalizability and external validity compared to RCTs [26, 27, 32].

RWE studies are complementary to RCTs in the generation of scientific evidence

The lack of generalizability of RCTs may contribute to a limited uptake of novel treatments despite positive evidence within RCTs [3, 26]. This may be due to uncertainty about how this evidence may transfer to broader patient groups and how to integrate these treatments into routine practice [10, 30].

In addition to a higher external validity, RWE studies have the potential to address a number of further limitations of RCTs. For example, RCTs often underestimate (long-term) toxicity and they rarely, or with a delay, explore certain research topics such as head-to-head comparisons of novel medications or interventions. Analyses with various clinical outcomes, in particular long-term and quality of life parameters are relatively infrequently addressed [10, 26, 32]. Moreover, a substantial number of RCTs focus on surrogate parameters instead of clinical parameters that are more clinically relevant [10, 32]. Therefore, RWE studies can be used in a supplemental manner to create surveillance for new therapies and enable analyses of differential benefits of therapies in routine clinical care or by patient subgroups [35]. Finally, RCTs are relatively time and resource-intensive [10, 29]. On the other hand, RWE studies have the promise of being conducted significantly faster and more resource-efficient but only once the necessary structures have been established in the centers and institutions. The financing sources for RWE studies are principally the same as for RCTs.

These critical remarks of caution do not intend to challenge the high value of RCTs, especially in the assessment of the efficacy of novel therapies. We believe, however, that RWE studies based on the data already captured in clinical systems can yield important additional insights into research and clinical care if they are being conducted at a high level of quality. To achieve this, we need sophisticated planning and careful execution to dispel a number of concerns about RWE studies. The large amount of apparently available electronic data may mislead researchers to conduct studies without elaborate attention to a stringent study design. This may include RWE studies that do not properly attend to data quality and thereby run the risk of biased data [19, 35]. This further includes RWE studies that conduct ‘data dredging’ in disregard of scientific principles [2] or RWE studies which are initiated with a view towards commercial objectives instead of clinical or scientific insights [14].

In principle, the limited internal validity of RWE studies, primarily due to the general lack of randomization, is an important criticism and urges towards caution [2, 17]. Certainly, the lower internal validity needs to be addressed to disentangle the effect of the treatment under investigation from other factors [3]. We will discuss later how advanced statistical techniques may support researchers in responding to this challenge.

Internal and external validity are both vital cornerstones of good science. While RCTs have higher internal validity, RWE studies have higher external validity. Thus, there may be a complementarity in the generation of scientific evidence [3, 29, 33].

For example, RWE studies can help in setting the research direction and in generating hypotheses of future RCTs or serve as the foundation for future confirmatory RCTs [29]. On the other hand, RWE studies can extend our knowledge of treatment effectiveness and safety by generalizing the findings of prior RCTs [29]. They may further describe underutilization of therapies or reveal overtreatment [3], and also foster research of rare tumors because they may allow the use of data sets with sufficiently large patient cohorts [13].

**Fig. 1:**
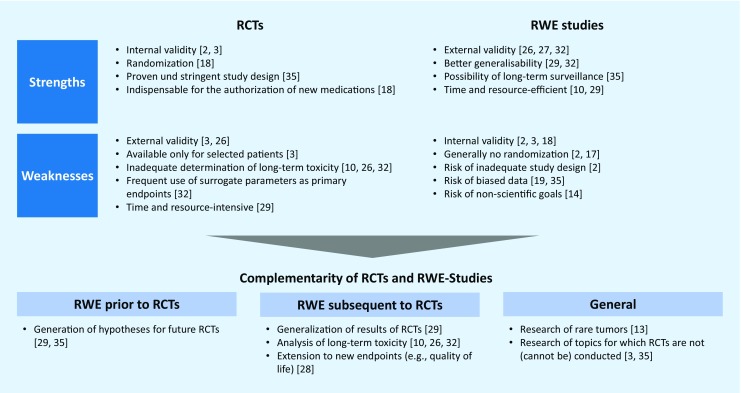
Strengths and weaknesses of RCTs vs. RWE studies, and their complementarity

By demonstrating that positive results of RCTs are also applicable in routine clinical practice RWE will also increase the confidence of oncologists with respect to their use of anti-cancer therapies in routine clinical care. Perhaps along the way they may uncover boundary conditions and derive adapted approaches and safety insights for subpopulations (Fig. 1).

## Concept and examples of RWE studies

A good example to demonstrate the complementarity of RCTs and RWE studies and the potential therein is a study recently published in the *Journal of Clinical Oncology* (JCO) [22]. The research question of this study resulted from disputed results of several international RCTs regarding the role of neoadjuvant chemotherapy (NACT) and primary cytoreductive surgery (PCS) in ovarian cancer of stages IIIC and IV. This study, which analyzed the comprehensive database of 1538 patients from 6 renowned American centers of the National Comprehensive Cancer Network (NCCN) found a survival benefit for patients in stage IIIC in the PCS group. This correlated with a subgroup analysis of a prior European Organisation for Research and Treatment of Cancer (EORTC) study and is in line with current treatment guidelines, e. g. in Germany, the Association of the Scientific Medical Societies in Germany (AWMF) S3 guideline. The comparability of both groups was ensured by means of a refined propensity score matching (*n* = 594). The general increase of NACT indications for ovarian cancer during the analysis period of this study should be viewed critically since the study also showed that interval cytoreductive surgery (ICS) did not improve outcomes. However, the study confirmed that NACT is noninferior to PCS in stage IV. Thus, several new research questions (hypotheses) with regard to optimizing the treatment algorithm for ovarian cancer in stages IIIC and IV may be derived from this RWE study.

Other examples for the complementarity of RCTs and RWE studies are long-term studies on treatment safety [6] or on topics for which RCTs were not feasible (especially for patients with a rare tumor) [23]. These examples demonstrate the direction that RWE studies could and should be taking. An increased level of data depth and data quality combined with stringent methods and processes should further reduce the limitations while increasing the quality and quantity of RWE studies [24].

## Benefits of establishing an oncology RWE network

There are a range of additional benefits from the establishment of a network of cancer centers that collaborate with each other. In a partner network, cancer centers can learn from each other and exchange experiences in topics such as building the required infrastructure, creating high-quality data sets, or the design and execution of the RWE studies. In addition, the cancer centers can collectively analyze their data to form sufficiently large patient cohorts. Moreover, a network of cancer centers would ideally have centers that partially employ different processes and regimens in the treatment of patients. It is this variation in practices that may potentially help uncover novel insights into as yet insufficiently covered factors and their impact on treatment effectiveness. Furthermore, treatment alternatives (whose pros and cons have not been fully defined in treatment guidelines) can be tested [19].

Such topics can currently not, or only partially, be derived from epidemiological [4] or clinical cancer registries [15] because these registries have a different main objective. RWE studies, as described in this article, are of course not completely new but rather based on established observational study methods such as cohort studies [16], registry studies [25] and population studies [20]. These registries and types of studies provide different and crucial insights into the quality of cancer care but capture data often with a time delay and necessarily in a limited depth. RWE studies are therefore also complementary to these efforts.

## Challenges and success factors

### Establishing a RWE network

In order to achieve the possibilities and objectives described above, several tasks need to be addressed. These refer predominantly to the current reality of fragmented clinical IT systems, the quality of the data therein, as well as to information governance and operations. Resolving these challenges prior to the initiation of studies will set the technical and operational infrastructure to conduct RWE studies more efficiently, more reliably, and at a consistently high level of quality. In this context, continuous attention and efforts to utilize appropriate technologies and an up to date management of the “big data sets” are of particular importance [13].

#### Identification of partner cancer centers

Establishing a network begs the question on how to identify suitable partner cancer centers for the network. The high importance of an appropriate technical infrastructure and especially of high data quality necessitates the inclusion of partner cancer centers that are committed to invest time, resources and determination into optimizing their data infrastructure. Furthermore, key personnel in the cancer centers should be convinced of the benefits of such a RWE network. Lastly, one should strive to connect cancer centers that may complement each other with regard to their patient profiles, research areas, and geographical variation. On this basis, the participating centers are better equipped to cover a wide range of influencing parameters, that may otherwise be neglected, and thereby also counteract potential confounders [18].

#### IT systems and databases

Experience tells us that clinical data sets currently frequently reside in ‘silos’ [27, 35]. In essence, the data are located in different domains because they are being captured by organizationally different clinical units, by means of different systems and are ultimately stored in different infrastructure units [9]. Hospitals and cancer centers typically do not have unified and integrated data warehouses. On the contrary, laboratory results are stored in a laboratory information system (LIMS), (radiological) imaging data in a picture archiving and communication system (PACS), prescription data in the pharmacy system etc. This fragmented landscape poses a challenge for any given study even within a specific type of tumor at a single cancer center. In addition, a highly effective RWE network should strive to achieve data comparability across the partner cancer centers to enable multicenter studies. Data, however, are often incomparable within a center and the comparability across cancer centers is thus even more challenging.

The commitment of decision makers in the cancer centers is essential

A key success factor in addressing these challenges is the construction of integrated data warehouses that collect, link and store all relevant data sets [9, 35]. This infrastructure should be built in a manner that ensures comparability of the data for the purpose of conducting research studies [33]. Technically, this can be facilitated by common standards of electronic data exchange such as HL7 and tools designed to ‘extract-transform-load’ (ETL). The data can thereby be extracted from a source, transformed into the desired format, and loaded into a target infrastructure. This process should be supported by medical ontologies and in practice by the diagnosing physicians and the treating oncologists. Eventually, this can result, under the leadership of specialized IT personnel, in a common data model, which will also greatly advance data comparability across cancer centers in the RWE network. Of course, tools for data protection and pseudonymization/de-identification of patient data need to be embedded in the technical solution.

Besides these technological requirements, an essential criterion for the success of such a project is the commitment of key decision makers in the cancer centers [13]. Some cancer centers have already started to build such solutions but clearly further efforts and advances are necessary in order to fully utilize the potential [27].

#### Data quality

Another important aspect is the quality of the data. The systems that routinely collect and store patient data have usually not been designed with the objective to eventually utilize the data for research purposes. Similarly, data entry into documentation systems is rather unpopular (not only) with physicians because it detracts from their direct patient contact by significantly limiting the available time for this. However, completeness and validity of the data collected in clinical routine care are of utmost importance for a meaningful and analytically ready RWE network [5, 29]. Specifically, there are four distinct yet related challenges: the completeness, common structure, and accuracy of the data as well as the availability of novel types of data.

Missing data is a common issue with health data in general [2]. This limits the size of the patient cohort that can be completely analyzed and may also introduce a bias into the data set that could potentially invalidate the findings [35]. Moreover, cancer patients are often treated by more than one department and unit within a center as well as by office-based oncologists. Cancer centers would need to strive to achieve a nearly complete follow-up to enable outcome research with a comprehensive and long-term view. It is obvious that the success of a high-quality RWE program depends on data sets that are nearly complete and in particular it requires that any incompleteness is not due to a systematic bias [34]. Within an institution, data completeness may be improved by a change in the front-end data capture coupled with illustrating the value of capturing full records. Across institutions, technology in the sense of common standards and interfaces may also contribute to the analytical integration of commonly collected data. In addition, the commitment among the decision makers and sufficient capacity of trained personnel are both instrumental in ensuring comprehensive follow-up together with partner institutions.

Some types of data in electronic medical records, such as anamnestic data, comorbidities or toxicity, are frequently recorded as free text instead of being stored as structured data variables [24]. One advantage of real-world data is the ability to construct data sets with long follow-up periods. The potential to fully utilize the theoretically available data is confined by historical, paper-based documents. Both challenges can be increasingly addressed by a mixture of technology and organizational measures. For example, technically by a change in the front-end systems requiring the structured input of key data points along with a general motivation of the staff of a cancer center [35]. Also, natural language processing (NLP) software solutions that utilize medical dictionaries (e. g. SNOMED, LOINC) can transform unstructured data both retrospectively and prospectively [12]. They should not be used in isolation but rather by medical coding teams.

Various reasons can endanger the accuracy of data. For example, data may be inaccurate due to an error creating or in entering patient data, or due to a change in classification schemes etc. Data accuracy can be fostered by a combination of electronic means of assuring quality, e. g., checking validity at the point of data entry or business quality rules, and periodic quality checks. Reviewing the distribution of data variables and conducting logic checks may uncover systematic inaccuracies.

Some, primarily scientific, types of data such as new biomarkers, genomics data, or novel laboratory tests are currently not systematically collected or in a heterogeneous manner. This applies also to patient-reported outcomes (PRO) such as structured assessments of patients’ quality of life. Increasingly, (psycho-) oncologists suggest that quality of life data should be key outcome parameters in oncology. The clinical results of novel therapies, unfortunately, still generate in many cases only marginal improvements in overall survival but may result in meaningful differences in patients’ quality of life [28]. Considering PRO data may also be helpful in uncovering symptomatic toxicities such as nausea or vomiting that are often captured incompletely [21]. A RWE network should therefore envisage the incorporation of these novel types of data systematically into their routine clinical practice [12]. Of particular relevance is the establishment of a user-friendly process that captures patients’ quality of life assessments [28].

#### Data privacy and protection

The fundamental importance and the indispensable cornerstones of the protection of patient data [5] have been recently reiterated by the (German) National Ethics Committee (Deutscher Nationaler Ethikrat) in a comprehensive report in the context of big data in healthcare [7]. In parallel, the new General Data Protection Regulation (GDPR) will come into effect in the European Union as of May 2018. This updated regulation further expands the scope of data privacy and protection by including new principles such as data protection and privacy by design that obligates the implementation of technical and organizational measures that secure patient data already at the design of systems [8]. Two legitimate claims oscillate here: the individual right to data privacy and the right of the population that improvements in cancer therapy may be developed based on data analyses.

Sophisticated solutions have been developed for the pseudonymization or de-identification of data. Various technical solutions are available to protect the data. They should be combined with well-established organizational processes and training of personnel. Furthermore, the data sets could stay within the confines of each cancer center and be analyzed only by staff associated with the cancer center. Multicenter studies could be conducted by means of federated data analysis in this scheme.

#### Operations

Integrating data from fragmented systems, ensuring high data quality and enhancing it further, while securing data privacy requires a strong governance framework within a cancer center. The framework needs to describe the decision rights and roles and responsibilities of the various departments within a cancer center and the hospital. This is not only crucial in the formation of the network but will also govern how RWE studies are to be conducted within a center and in partnership with other centers. All requirements and tasks described above also necessitate a sufficient capacity of dedicated, specialized personnel [5]. Only this interplay can enable a high level of scientific and methodological rigor (Table 1).

**Table 1:** Challenges and success factors in establishing a RWE structure in cancer centers

Categories	Elements	Challenges	Success factors
Network	Identification of partner cancer centers	Centers need to be interested in modern IT and high-quality data	Identify partner centers with the vision to advance their IT/data infrastructure
		Centers need to be interested in conducting high-quality research	Identify partner centers with a strong research profile and broad study activities
IT	Fragmented systems and databases	Data generated by different departments (pathology, radiology, pharmacy) and stored there	Build integrated data warehouses utilizing technologies, such as HL7 or ETL, supported by medical ontologies and a common data model
		Different data sources and documentation systems with missing or difficult harmonization	Ensure organizational commitment to pool the data from disparate infrastructures into a joint data warehouse
		Missing or heterogeneous quality standards	
Data quality	Data completeness	Some data types are frequently incomplete (e.g., toxicities)	Improve systems for data capture within institutions and convince staff of the importance of capturing full records
		Patients frequently treated across institutions	All institutions utilize common interfaces for data exchange and ensure sufficient capacity of trained personnel
		Sometimes no a priori homogeneous and stringent data capture (anamnesis, quality of life)	
	Data structure	Data captured in free text, e.g., comorbidities and disease history	Change front-end data capture to structured variables
		Data not captured in EMR but only on paper	Utilize natural language processing and medical coders
		Data captured in free text, e.g., comorbidities and disease history	
	Data accuracy	Errors in creating or entering data; measurement errors	Employ electronic means of quality assurance
		Updated classification schemes may lead to inaccuracy in retrospective data	Conduct periodic quality checks by team of data scientists and oncologists
			Re-classify data where needed (by oncologists/pathologists)
	Novel types of data	New biomarkers or laboratory tests not yet recorded	Introduce novel data points in routine data capture
		Patient-reported outcomes (e.g. quality of life) not captured	Implement the collection of patient-reported outcomes (e.g. quality of life) into routine data capture
Information governance	Data privacy and protection	Strict protection of patient data is paramount and mandated by regulation (e.g. National Ethics Committee, EU GDPR)	Conduct pseudonymization/de-identification of the data
		Inadequate systems, processes, as well as human errors may lead to a data breach	Employ data protection tools, build strong organizational processes, and train personnel
Operations	Governance framework	Within a center and hospital the decision rights, roles and responsibilities need to be defined	Build a strong center governance to manage the RWE study infrastructure within a center
		The network level further necessitates an appropriate governance across centers	Establish a network governance that regulates the collaboration of centers in the network
	Specialized personnel in medical informatics	Implementation requires dedicated, multi-professional team and a specialist skill set in medical informatics	Collaboration within the multi-professional team

*IT *information technology, *EMR* electronic medical records, *EU GDPR* European Union General Data Protection Regulation, *RWE* real word evidence

### Conducting RWE studies

For high-quality RWE studies there are additional requirements with respect to design, management, and publication.

#### Study design and execution

A critical review of phase IV trial protocols suggest that many of them neglect to account for recognized measures of quality assurance in the design of these studies [14]. A clinically relevant research question needs to be postulated based on a stringent medical theory and the corresponding hypotheses need to be established. These may then be analyzed with an appropriate and sufficiently large dataset and by applying suitable methods [1]. This necessitates a substantial medical expertise in oncology from the inception of the study [29]. Therefore, RWE studies should be conducted hand in hand with stakeholders who possess the requisite clinical and biometric expertise and who design and execute the studies independently under primarily scientific aspects [2].

The tasks require sufficient and dedicated specialized personnel

Real-world data are vulnerable to a range of biases [35]. These include selection bias, information bias, measurement error, confounding, and Simpson ’s paradox [11], as well as performance, detection or attrition bias [35]. It is thus necessary to employ stringent methods to assess and ascertain the data quality, for example, by integrating the Cochrane risk-of-bias approach [9].

As discussed above, the key limitation of RWE studies is their lower internal validity [2, 3]. In contrast to RCTs, and more generally, studies with an experimental design, RWE need to apply methods that single out the effect of the treatment under investigation [27]. To this end, there are a number of statistical matching techniques, such as propensity score matching, inverse probability weighting, or stratification [17]. The propensity score method has some parallels with controlled trials on some levels [17]. Conceptually, the method seeks to analytically generate a control group that resembles the treatment group very closely with respect to the characteristics of the patient groups and other impact factors.

#### Publication of RWE studies

A frequent criticism of post-marketing studies, which include RWE studies, is the partial practice of opaque reporting of findings and selective publication [2]. A recent *British Medical Journal* (BMJ) article reported that only a small proportion of post-marketing studies were published in scientific journals [31]. This development is problematic because it does not contribute to scientific progress and contravenes common principles of good scientific practice. This raises concerns about the motivation for and the scientific discussion of post-marketing studies. A general obligation to publish (completed and discontinued) RWE studies conducted in an oncological RWE network should already be agreed upon in the planning phase of a study [2]. The publications should be transparent with respect to the original research question, the design of the study, its analysis and interpretation ([35]; Table 2).

**Table 2:** Challenges and success factors in conducting RWE studies

Categories	Elements	Challenges	Success factors
Study Design	Principles of good science	Some RWE studies deviate from established standards of good science and engage in ‘data dredging’	Adhere to principles of the philosophy of science, i.e. based on theory, derive hypotheses and test these
	Oncology expertise	Researchers without oncology expertise and a proper understanding of the clinical care setting conduct studies	Involve oncologists with the requisite medical expertise closely in the study design and overall research process
	Biased data	Routinely collected data vulnerable to a range of biases	Apply stringent methods to assess and ascertain data quality
		These may render results unreliable if unaddressed	Apply appropriate study designs and statistical analysis plans
	Causality	Lower internal validity is a key limitation of RWE studies	Utilize advanced statistical methods such as propensity score matching to create comparable control groups
			Consider existing causal relationships already in the study design
	Specialized personnel in data science	The methodological challenges inherent in RWE require specialist personnel in data science	Recruit and collaborate with specialist personnel in data science
Study publication	Publication	Selective publication of results does not foster scientific insight and trust in RWE studies	Commit to publish all RWE studies in the public domain with transparency on design, analysis, and interpretation of results

*RWE* real word evidence

## Conclusion

- “Big data” can be considered the material basis for the realization of RWE studies. These may be a complementary partner of RCTs and thereby a valuable tool in clinical research in oncology.
- Modern IT concepts and technologies enable the digital and structured capture of complex oncological information in addition to routine medical data.
- Thereby, it is possible to analyze data longitudinally and data that has been collected with different methods.
- The individual centers in a RWE study network may conduct national or international benchmarking, depending on the composition of the network, in addition to analyzing their internal clinical context.
- This may not only yield clues about outcomes of current treatment pathways but also about alternative approaches or about new research-related hypotheses.
- RWE studies are therefore a meaningful complement to RCTs which typically analyze pre-selected patients and to clinical registries which usually operate on a reduced data scope.
- The ambition to conduct high-quality RWE studies in oncology poses significant challenges for all stakeholders with regards to IT, personnel, organizational, financial, and data privacy aspects.
- These challenges can only be overcome jointly in order to achieve the legitimate aim of a relevant improvement of quality, effectiveness and safety in oncological care.
